# Pro‐inflammatory cytokine IL‐21 correlates with the reactive oxygen species and 25‐hydroxy vitamin D in rheumatoid arthritis patients

**DOI:** 10.1002/iid3.1308

**Published:** 2024-07-26

**Authors:** Ma Shufang, Han Xiaojiao, Kang Yinhong

**Affiliations:** ^1^ Rheumatology and Immunology Department Fourth Central Hospital of Baoding City Baoding Hebei Province China; ^2^ Obstetrics Department Fourth Central Hospital of Baoding City Baoding Hebei Province China

**Keywords:** interleukin 21, reactive oxygen species, rheumatoid arthritis, rheumatoid factor, vitamin 25(OH)D

## Abstract

**Background:**

Rheumatoid arthritis (RA) is a chronic autoimmune disorder and its characteristics include the immune system's invasion of the healthy lining of the joints and the articular structures degeneration. The IL‐21 pro‐inflammatory cytokine, and the reactive oxygen species (ROS) might have a role in the RA etiopathogenesis. The present study assessed the correlation of IL‐21 with vitamin 25(OH)D and the ROS.

**Methods:**

The study included 120 RA patients and 60 healthy group. The RA patients were categorized based on rheumatoid factor (RF) seropositivity or seronegativity and the RA severity. Chemiluminescent immunoassay and 10% hematocrit were used to check levels of vitamin 25(OH)D and ROS, respectively. ELISA was used for the detection of IL‐21 in the plasma.

**Results:**

The RA patients had a significantly reduced vitamin 25(OH)D level compared to the healthy controls. The IL‐21 and ROS were however significantly increased in the RA patients compared to the controls. Further, the seropositive RF and the high RA severity patients had significant IL‐21 and ROS increase in comparison with the seronegative RF and the low severity RA patients. Finally, IL‐21 negatively correlated with vitamin 25(OH)D, but positively correlated with the ROS.

**Conclusion:**

This is the first investigation to confirm the relationship between IL‐21 with vitamin 25(OH)D and the ROS among the RA patients. The findings indicate that vitamin 25(OH)D is reduced in the RA patients' serum. ROS and IL‐21 are also associated with increased RA severity.

## Graphical abstract

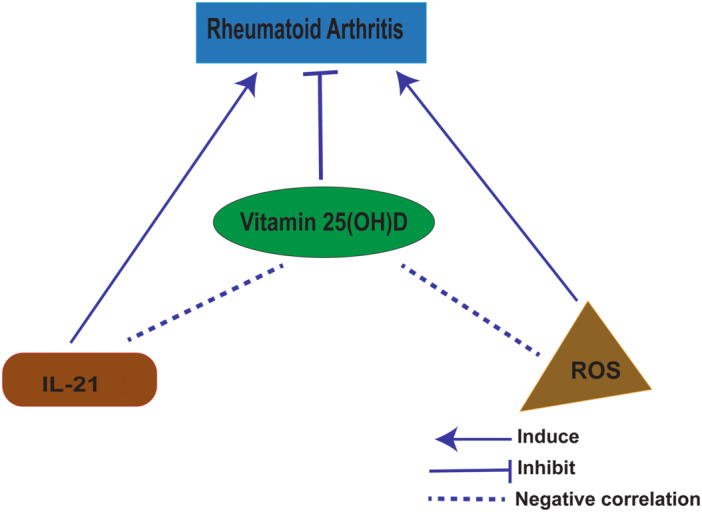

## 1 INTRODUCTION

Rheumatoid arthritis (RA) is defined as a chronic autoimmune illness characterized by the chronic synovial inflammation and joints destruction.^1^ RA is linked to several immunological dysregulations, including elevated activated T‐lymphocytes numbers and aberrant inflammatory cytokines expressions. The several inflammatory‐associated cytokines produced by infiltrating B, T cells, and macrophages in the tissues and synovial tissues collectively lead to joint inflammation,^2^ and have significant function in the joints damage and inflammation propagation in RA.^3^ Autoimmune disorders are linked to the immune system aberrant response and elevated immune modulators expression might enhance the inflammatory cells activation and survival, thus facilitating the autoimmune diseases development.^4^

RA etiopathogenesis remains incompletely clarified and various mechanisms have been implicated in its development. Besides the involvement of 25‐hydroxy vitamin D (vitamin 25(OH)D) in the metabolism of bone and calcium, it has been reported as an essential environmental factor relevant to various autoimmune disorders, such as RA.^5^ 25(OH)D has an immune‐modulatory role and its increased consumption is linked to reduced RA risk. In addition, vitamin D regulates the redox signaling pathway hence control the formation of reactive oxygen species (ROS).^6^ Further, the vitamin D checks inflammation by regulating adaptive and innate immunity.^7^

Activated immune cells and synovial fibroblasts lead to increased inflammatory cytokines production, which has a crucial function in the progression and development of RA.^8^ The RA was earlier hypothesized as Th1‐cell‐mediated autoimmune disorder. However, T helper 17 (Th17) cells are currently also regarded as essential in the RA disease process.^9^ The RA inflammation characteristic occurs as a result of the inflammation‐abundance‐promoting cytokines over the inflammation‐inhibiting cytokines. Among the various pro‐inflammation cytokines, interleukin (IL)‐21 is an IL‐2 cytokine family member belonging to the type 1 family, which is primarily produced by the natural killer and CD4^+^ T cells.^10^ The IL‐21 plays several pleiotropic roles, by controlling both the innate and the adaptive immune responses.^11^ Consequently, IL‐21 exert critical functions in regulating and directing B‐ and T‐cell response, eventually causing the antibodies formation.^12^

ROS play a role as the second messenger in the nuclear factor‐κB (NF‐κB) activation thus permitting various inflammatory mediators’ transcription.^13^ The ROS hyperproduction leads to the biomolecule's oxidation. According to literature, there is an occurrence of antioxidant defense system weakening and subsequent oxidants overproduction, hence resulting in the oxidative stress among autoimmune disease patients.^14^ Consequently, there is an occurrence of a complex interplay between mediators of inflammation and ROS. No study has evaluated the interplay between vitamin D mediators, the inflammatory cytokine, and ROS, and whether they might have a joint role in the establishment or control of RA. The present study aimed to evaluate the vitamin 25(OH)D, ROS, and IL‐21 cytokine levels in the RA patients’ blood, and to determine the association of IL‐21 with ROS and vitamin 25(OH) D. These described parameters were additionally compared in patients and further categorized on the basis of the severity of the disease and the rheumatoid factor (RF) presence or absence.

## 2 MATERIALS AND METHODS

### 2.1 Patients

**Figure 1:**
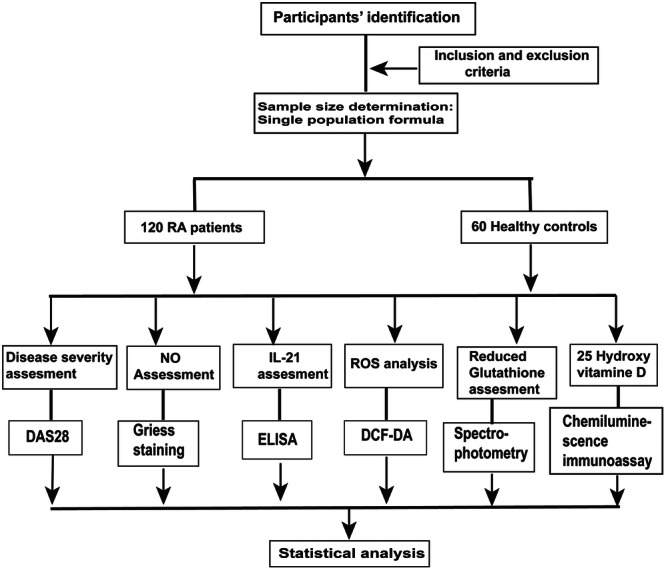
Experimental design flow diagram. DAS28, 28‐Joint Count disease activity score; DCF‐DA, 2′,7‐Dichlorofluorescein diacetate; ELISA, enzyme‐linked immunosorbent assay; IL‐21, interleukin 21; NO, nitric oxide; RA, rheumatoid arthritis; ROS, reactive oxygen species.

The investigation was done on 120 RA patients between 24 and 60 years, who were visiting orthopedics department of the Fourth Central Hospital of Baoding City. Patients included in the investigation were all newly diagnosed cases who met the 2010 European League Against Rheumatism RA classification procedure. The control arm was made of 60 healthy people between 23 and 60 years, and the sex‐matched people, as shown in Figure 1. The research was done between August 2021 and February 2022. Informed consent was obtained from all the study participants. The study sample size was derived through a single population ratio formula by assuming that the proportion of pregnant RA patients in Fourth Central Hospital of Baoding City was 8%. The precision and confidence interval were 5% and 95% (*Zα*/2 = 1.96), respectively. All the blood donors provided a written consent before the blood withdrawal. The research approval was obtained from the Ethical Review Committee of the Fourth Central Hospital of Baoding City and all methods were performed in accordance with the relevant guidelines and regulations. The exclusion criteria were patients suffering from any form of chronic illness, alcoholics, or smokers. The study blood donors were not under any form of vitamin supplement. The features of the RA patients and the controls were presented in Table 1.

**Table 1:** The baseline characteristics of the RA patients versus the healthy groups presented as mean ± SD.

Parameter	RA	Control	*p*
Age (years)	43.07 ± 6.46	44 ± 5.33	.21
Weight (kg)	61.21 ± 4.32	60.41 ± 3.14	.62
Height (cm)	166.05 ± 5.03	164.24 ± 3.63	.77
ESR (mm/h)	31.09 ± 7.25	14.09 ± 4.06	.001*
BMI (kg/m^2^)	27.53 ± 2.36	26.89 ± 6.11	.58
NO (µM)	52.24 ± 1.42	22.33 ± 7.26	.001*
GSH (mM)	42.09 ± 1.42	10 ± 5.02	.001*

Abbreviations: BMI, body mass index; ESR, erythrocyte sedimentation rate; GSH, glutathione; NO, nitric oxide; RA, rheumatoid arthritis.

* *p* < .05.

### 2.2 Measurement of disease severity

The determination of RA severity was done through the calculation of 28‐Joint Count disease activity score (DAS28). The criteria utilize tender and swollen joints numbers, erythrocyte sedimentation rate (ESR) together with the health assessment of the patient through the use of Visual Analogue Scale. The DAS28 ≤ 3.2, the DAS28 > 3.2 ≤ 5.1, and the DAS28 > 5.1 corresponds to low, moderate, or high RA severity, respectively.

### 2.3 Blood samples

The patients and the control group's blood were drawn into various anticoagulants vials. Cytokine estimation was done using plasma. The buffy coat was then removed after the plasma storage and erythrocytes washing was done thrice with the use of phosphate buffered saline (PBS), (pH 7.2). ROS quantification was done using 10% hematocrit. Hemolysates were prepared as described elsewhere.^15^ Briefly, lysis of cells was done using 10 volume of the chilled isotonic saline for 2 h, pH 7.4. The samples were centrifuged at 4°C and 3000 rpm to obtain the hemolysates. Later, the storage of the hemolysates were done at −20°C for further analysis and estimation of glutathione (GSH).

### 2.4 Vitamin 25(OH)D assessment

The vitamin 25(OH)D level was estimated from the freshly obtained serum samples of RA patient, as previously reported.^16^ Total vitamin 25(OH)D) in serum was estimated using direct competitive chemiluminescence immunoassay in a Liaison auto‐analyzer (Liaison, DiaSorin). As instructed by the manufacturer, the 25(OH)D serum concentrations of approximately <10, <30, and 30–100 ng/mL were considered to be severe deficiency, Vit D insufficiency, or normal Vit D, respectively.

### 2.5 Synovial fluid (SF) isolation and culture

SF was obtained from the RA patients and collected in a 15 mL sterile falcon tubes containing anticoagulants. The samples in the tubes were diluted twice using sterile PBS and centrifuged for 10 min at 400*g*. The pellets were resuspended in a complete Dulbecco's modified Eagle media supplemented with 10% fetal bovine serum, penicillin (100 U/mL), and streptomycin (100 mg/mL). The cells were seeded in T‐25 flasks and incubated at 37°C with CO_2_ (5%). Fresh media was added to the cells after 48 h of incubation. The culture media was routinely refreshed after every 3 days.

### 2.6 ROS determination

Production of intracellular ROS was assessed using the 2′,7‐Dichlorofluorescein diacetate (DCF‐DA) as clarified elsewhere.^17^ In brief, SF were plated in a sterile 12‐well plates, washed using 10 mmol/L HEPES‐modified Tyrode's (HT) solution, 37°C, pH 7.4, and eventually loaded using 10 mmol/L of H2‐DCF‐DA dissolved using HT solution, 10 mmol/L of stock solution dissolved in dimethylsulfoxide (DMSO) or 2 mmol/L of H2‐calcein‐AM dissolved using HT solution, 1 mmol/L of stock solution dissolved in DMSO in the final volume of 500 mL for 20 min. Cells were later washed two times and incubated in the dark at 37°C using 300 mL of HT solution. The supernatant with the leaked calcein and DCF (extracellular) was later removed then stored in ice. Cells’ washing was done once and later lysed by adding 300 mL of distilled water. Extracellular and the intracellular fraction fluorescence was determined in a microplate reader.

### 2.7 NO assessment

The production of NO in plasma was measured through Griess staining procedure as described elsewhere.^18^ In brief, 50 μL of cell supernatants were introduced in the 96‐well plates. Next, same amounts of the Griess reagent (Beyotime) were added into the wells following the manufacturer's guidelines. Plates were eventually incubated in a room temperature. The standard used was sodium nitrite reagent and the reaction was determined with a microplate reader (Thermo Fisher 1510) at OD of 540 nm. Reporting of data was done as the mean values from experiments repeated thrice.

### 2.8 Reduced GSH assessment

GSH in the hemolysate was determined as described elsewhere.^19^ In brief, the hemolysate was deproteinized by adding an equal amount of 4% sulfosalicylic acid and then centrifuged. Later, 0.5 mL of the supernatant was added to Ellman's reagent (bis‐(3‐carboxy‐4‐nitrophenyl) disulfide) (4.5 mL). The reduced glutathione was equivalent to absorbency (at 412 mU). To estimate the oxidized glutathione, additional hemolysate aliquot was incubated using glutathione reductase for 10 min at 37°C. The protein was later precipitated and glutathione expression was determined. The difference between the reduced and oxidized glutathione was used in estimating the oxidized glutathione concentration in the hemolysate.

### 2.9 Assessment of IL‐21

The serum concentration of IL‐21 was determined using a commercial enzyme‐linked immunosorbent assay (ELISA) kit (R&D) as per the guidelines of the manufacturer. The ELISA reader (Thermo Fisher Scientific) was used to determine the absorbance at a primary wavelength of 450 nm. The cutoff (detection limit) was set to 7.2 pg/mL as stipulated by the manufacturer.

### 2.10 Statistical analysis

Representation of data was done in the form of mean ± SD Distribution of data was assessed with Shapiro–Wilk test. Difference between the two groups were investigated by Student's *t* test or Mann–Whitney test, whereas difference between the three groups were assessed using analysis of variance, where appropriate. Spearman's rank correlation test was used in determining the correlation of IL‐21 with vitamin 25(OH)D and ROS. The results were considered as statistically significant when *p* < .05.

## 3 RESULTS

In total, this investigation enrolled 120 RA patients (63 males and 57 females, age 43.07 ± 6.46 years) and 60 healthy group (32 males and 28 females, age 44 ± 5.33 years). The mean weight of the RA patients was 61.21 ± 4.32, whereas the control group weighed 60.41 ± 3.14 kg. The mean height of the RA and the control groups were 166.05 ± 5.03 and 164.24 ± 3.63 cm, respectively. The body mass index between the RA and control groups were 27.53 ± 2.36 and 26.89 ± 6.11, respectively. The mean ESR in the RA patients was 31.09 ± 7.25 mm/h, whereas the mean ESR in the control group was 14.09 ± 4.06 mm/h (*p* = .001). Similarly, the mean NO levels were 52.24 ± 1.42 and 22.33 ± 7.26 µM, whereas the mean of GSH were 42.09 ± 1.42 and 10 ± 5.02 mM in the RA and the control groups, respectively, as shown in Table 1.

**Figure 2:**
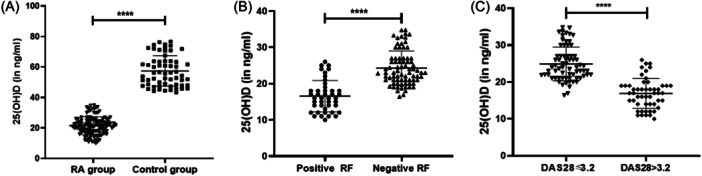
Assessment of the 25‐hydroxy vitamin D (vitamin 25(OH)D) serum levels in the rheumatoid arthritis (RA) patients and the control group (A), the seropositive and the seronegative patients (B), and the 28‐Joint Count disease activity score (DAS28) ≤ 3.2 and the DAS28 > 3.2 patients (C). ****p < .00005.

The level of vitamin 25(OH)D was assessed from freshly separated RA patients’ serum samples. According to the findings, the RA patients demonstrated a significantly inhibited vitamin 25(OH)D level (21.49 ± 5.877 ng/mL) compared to the control group (57.39 ± 10.05 ng/mL) (*p* < .0001) (Figure 2A). Further, the expression of vitamin 25(OH)D level was assessed in the positive and negative RF patients. According to the observations, there was a significantly reduced vitamin 25(OH)D expression in the positive RF patients (16.53 ± 4.317 ng/mL) compared to the negative RF patients (24.26 ± 4.701 ng/mL), (*p* < .0001) as shown in Figure 2B.

Finally, vitamin 25(OH)D level assessment was done based on the severity of RA among the patients. According to the results, reduced vitamin 25(OH)D level was reported in the DAS28 > 3.2 group (16.9 ± 4.056 ng/mL). However, a significantly higher vitamin 25(OH)D level was observed in the low‐disease severity patients (DAS28 ≤ 3.2) (24.89 ± 4.566 ng/mL), as shown in Figure 2C (*p* < .0001).

**Figure 3:**
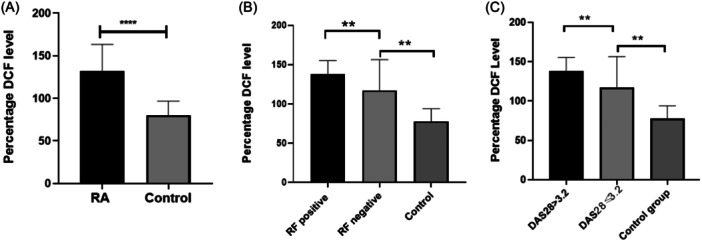
(A) Bar graph indicating the percentage expression of 2′,7‐Dichlorofluorescein (DCF) in the rheumatoid arthritis (RA) patients and the control group. (B) Expression of percentage DCF in seropositive, the seronegative RA patients, and the control group. (C) Assessment of the percentage DCF expression in the 28‐Joint Count disease activity score (DAS28) ≤ , DAS28 > 3.2, and the control group. **p < .005, ****p < .00005.

Next, intracellular ROS was determined by the assessment of the DCF‐DA expression in the RA and control group. The results confirmed a significant elevation of ROS in the RA group (132.1 ± 31.06 ng/mL) compared to the control group (80.31 ± 16.36 ng/mL) (*p* < .0001) as shown in Figure 3A. The level of ROS was then compared in the seropositive and seronegative patients. The results confirmed significant increase of the ROS expression in the seropositive patients (137.8 ± 17.62 ng/mL) compared to the seronegative (117.3 ± 39.11 ng/mL) and the control groups (77.96 ± 16.07 ng/mL), as shown in Figure 3B (*p* < .0001). In terms of disease severity, DAS28 > 3.2 patients demonstrated significantly increased level of ROS (137.8 ± 17.62 ng/mL) compared to DAS28 ≤ 3.2 group (117.3 ± 39.11 ng/mL) and the control group (77.96 ± 16.07 ng/mL), as shown in Figure 3C (*p* < .0001). We also confirmed a significant upregulation of ROS in the seropositive and DAS28 > 3.2 patients compared to the control group, 136.35 ± 15.43 versus 77.96 ± 16.07 ng/mL, *p* < .0001 (data not shown).

**Figure 4:**
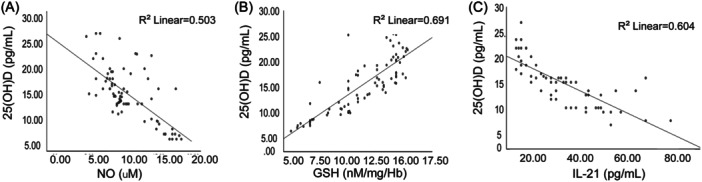
Analysis of the correlation between (A) 25‐hydroxy vitamin D (vitamin 25(OH)D) and nitrogen oxide (NO); (B) vitamin 25(OH)D and glutathione (GSH); and (C) vitamin 25(OH)D and interleukin 21 (IL‐21).

We then did a Spearman analysis to determine the correlation of IL‐21, NO, and vitamin 25(OH)D. According to our observations, there was a negative correlation between vitamin 25(OH)D and NO (*R* ^2^ linear = .503), as shown in Figure 4A. A positive correlation was confirmed between the 25(OH)D and GSH (*R* ^2^ linear = .691), as shown in Figure 4B. Finally, a negative correlation was observed between the 25(OH)D and IL‐21 (*R* ^2^ linear = .604), as shown in Figure 4C.

**Figure 5:**
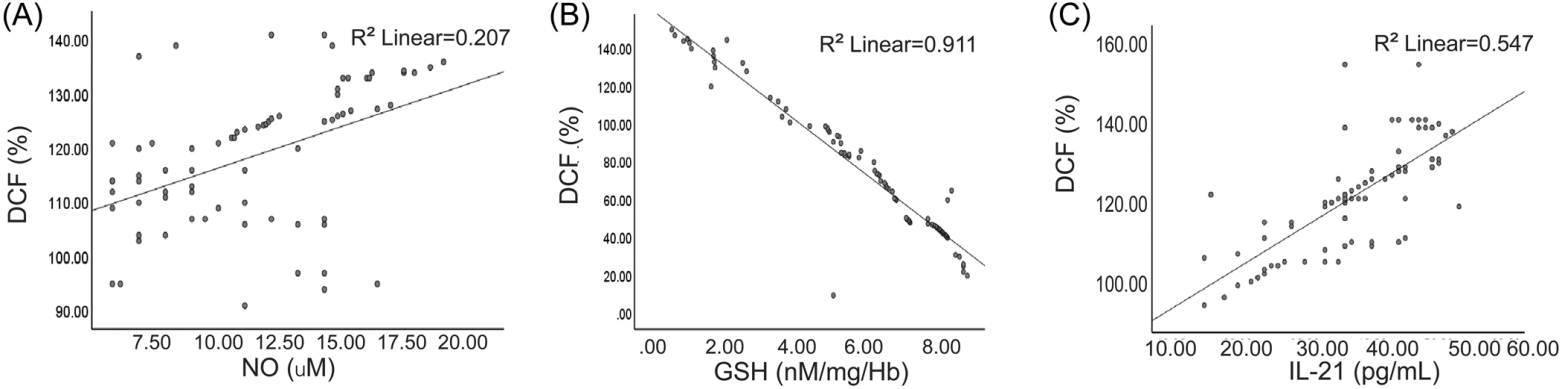
Investigation of the correlation between (A) reactive oxygen species (ROS) and NO; (B) ROS and glutathione (GSH); (C) ROS and interleukin 21 (IL‐21).

The association between ROS and NO, GSH, and IL‐21 was also determined through Spearman correlation. According to the results, there was a positive correlation between ROS and NO (*R* ^2^ linear = .207) as shown in Figure 5A. However, ROS was negatively associated with GSH, as shown in Figure 5B (*R* ^2^ linear = .911). Finally, analysis of ROS and IL‐21 confirmed a positive correlation (*R* ^2^ linear = .547), as shown in Figure 5C and Table 2.

**Table 2:** Correlation assessment of plasma levels of various biochemicals, 25(OH)D, and ROS.

Cytokine	25(OH)D	ROS
IL‐21	−0.604*	0.547*
NO	−0.503*	0.207*
GSH	0.691*	−0.911*

Abbreviations: 25(OH)D, 25‐hydroxy vitamin D; GSH, glutathione; IL‐21, interleukin 21; NO, nitric oxide; ROS, reactive oxygen species.

* *p* < .05.

## 4 DISCUSSION

Different biochemical factors or cytokines have been reported to have an effect on the progression of RA. Unfortunately, investigations reporting the interplay between ROS, 25(OH)D, and an inflammatory cytokine IL‐21 in RA are quite rare. The current work investigated the association between pro‐inflammatory cytokine IL‐21 with the ROS and vitamin 25 (OH)D in RA patients. We report significantly reduced vitamin 25(OH)D level among the RA and the positive RF patients, and reduced vitamin 25(OH)D level was reported in the DAS28 > 3.2 group. We also confirmed increased ROS expression in the seropositive and DAS28 > 3.2 patients. Negative correlation was observed between vitamin 25(OH)D and NO, and between the 25(OH)D and IL‐21. However, 25(OH)D and GSH were positively correlated.

In the recent years, various reports have confirmed that cytokines have essential functions in RA progression and pathogenesis. IL‐21 is a multifunctional cytokine mainly produced by the Th17, follicular helper T (Tfh), and the natural killer cells.^20^ IL‐21, which act on both nonlymphoid and lymphoid cells and have a role in the humoral immune responses’ regulation, is essential in the alterations to the T‐helper cells such as B cell subsets (such as naive B, memory B, and antibody‐secreting cells), Tfh, and regulatory T cells, and Th17, whose pathogenesis has also been clarified in several autoimmune disorders and linked with the elevated IL‐21 or IL‐21R expressions.^21^

Even though a previous study reported increased IL‐21 messenger RNA or IL‐21 protein levels in serum, impaired tissues, and peripheral blood mononuclear cells from autoimmune diseases patients and its correlation with the initiation and development of diseases like RA,^22^ this is the first investigation to report the interplay between IL‐21 cytokine, vitamin 25(OH)D, and ROS in the progression of RA among patients. IL‐21 expression was confirmed to be elevated in the seropositive RF and the high RA severity patients compared to the seronegative RF and low RA severity group.

The vitamin 25(OH)D play an immunomodulatory role through the intra‐cytoplasmic receptor interaction, thus posing a significant effect to the immune cells. In addition, the vitamin 25(OH)D has a role in the maintenance of the minimal level of ROS in the human body. Furthermore, recent investigations have shown a significantly reduced vitamin 25(OH)D level, with its drop being more notable among the high severity RA patients. According to the previous reports, the vitamin 25(OH)D regulates pro‐inflammatory cytokines release, including IL‐2, interferon‐γ, and tumor necrosis factor‐α.^23^

The reduced vitamin 25(OH)D level might induce inflammation among the patients with RA. Vitamin 25(OH)D role in redox signaling additionally indicate that reduced vitamin 25(OH)D elevates the formation of ROS.^24^ The formed ROS reacts together with the nitric oxide, hence leading to significantly heightened reactive peroxynitrite formation, which eventually results to the antioxidant's deletion and biomolecules oxidation. The consequence is a disturbed redox balance and an activated NF‐κβ signaling cascade, which eventually leads to the exacerbation of cytokine formation in RA patients.^25^

The IL‐21 cytokine and ROS overproduction have a role in autoimmune disorders, such as RA progression and development. ROS enhance biomolecules (DNA, lipids, and proteins) oxidation in the blood and SFs of patients suffering from RA.^26^ In addition, the reduced antioxidant levels have been reported among patients with RA, which are consequently responsible for the heightened production of ROS. The lower antioxidant levels also enhance hyaluronic acid depolymerization, hence leading to reduced viscosity of the joints and the elevated resorption of bones. According to the results of this current study, ROS overproduction leads to the increased RA severity, as conformed by the increased levels of ROS in DAS28 > 3.2 RA patients. Increased level of ROS in the seropositive RF patients demonstrates the RF's role in elevating the severity of RA by enhancing the reactive intermediates formation.

Increased IL‐21 levels reported among the DAS28 > 3.2 patients confirms that the IL‐21, together with ROS have a role in upregulating RA severity through oxidative stress promotion and inflammation. Furthermore, RF presence has a role in the exacerbation of the RA disease condition, as conformed by the increased level of IL‐21 cytokine in the seropositive RF compared to the seronegative RF patients.

The limitation of our investigation is that it lacks an analysis to address the disparities in NO and GSH levels between the RF‐positive and RF‐negative groups, as well as between the DAS > 3.2 and <3.2 groups. Further, our work did not show possible association of vitamin 25(OH)D, NO, and other numerous pro‐inflammatory cytokines. Consequently, future investigations are required to clarify the association of other pro‐inflammatory cytokines with vitamin 25(OH)D and NO.

## 5 CONCLUSION

The nobility of our investigation lies on the fact that it is the first to clarify IL‐21 level correlation with NO and vitamin 25(OH)D among the RA patients. Our study concludes that increased ROS and suppressed vitamin 25(OH)D are important for the elevated IL‐21 production. Finally, targeting the modulation of IL‐21 and ROS might be a noble approach in the regulation of RA pathogenesis.

## AUTHOR CONTRIBUTIONS

All authors contributed to the study conception and design. Ma Shufang and Han Xiaojiao did the material preparation, data collection, and analysis. Kang Yinhong wrote and revised the manuscript, and all authors commented on previous versions of the manuscript. The final manuscript was read and approved by all the authors.

## CONFLICT OF INTEREST STATEMENT

The authors declare no conflict of interest.

## ETHICS STATEMENT

The ethical approval for this study was obtained from the Ethical Review Committee of the Fourth Central Hospital of Baoding City.

## Data Availability

The data supporting the findings of this study have all been included in this submission. However, should the raw data be needed, the authors are willing to provide after a written request to the corresponding author.
